# Factors Associated with Having both Male and Female Recent Sexual Partnerships Among Men Who Have Sex with Men in Harare and Bulawayo, Zimbabwe

**DOI:** 10.1007/s10461-023-04262-2

**Published:** 2024-01-18

**Authors:** Morgan Davis, Godfrey Musuka, Munyaradzi P. Mapingure, Avi Hakim, Lauren E. Parmley, Owen Mugurungi, Innocent Chingombe, Sophia S. Miller, John H. Rogers, Matthew R. Lamb, Chesterfield Samba, Tiffany G. Harris

**Affiliations:** 1 Department of Epidemiology, Mailman School of Public Health, Columbia University, New York, NY USA; 2 ICAP at Columbia University, Harare, Zimbabwe; 3 Division of Global HIV and TB, Center for Global Health, US Centers for Disease Control and Prevention, Atlanta, GA USA; 4 ICAP at Columbia University, New York, NY USA; 5 AIDS and TB Programme, Zimbabwe Ministry of Health and Child Care, Harare, Zimbabwe; 6 Division of Global HIV and TB, Center for Global Health, US Centers for Disease Control and Prevention, Harare, Zimbabwe; 7 GALZ, Harare, Zimbabwe

**Keywords:** Men who have sex with men, Zimbabwe, Sexual and Gender Minorities, HIV, Sub-Saharan Africa

## Abstract

To better understand male and female sexual partnerships among men who have sex with men (MSM), we used data from a 2019 biobehavioral survey among MSM in Harare and Bulawayo, Zimbabwe to conduct bivariate analyses and multivariable logistic regression to determine whether sociodemographic characteristics and HIV-related factors were associated with having both male and female sexual partnerships within the last 6 months. Of included MSM (*N* = 1143), 31% reported both male and female partnerships in the last 6 months. Being married/cohabiting (adjusted odds ratio (aOR) = 8.58, 95% confidence interval (CI) = 4.92–14.95) or separated/divorced/widowed (aOR = 1.96, 95% CI = 1.24–3.08) vs. being single, and hazardous alcohol consumption (aOR = 1.58, 95% CI 1.19–2.09) were associated with higher odds of having both male and female recent partnerships. Being aged 35 + vs. 18–24 (aOR = 0.50, 95% CI = 0.31–0.81), condomless receptive anal intercourse at last sex with the main male partner (aOR = 0.43, 95% CI = 0.26–0.74), and positive HIV status (aOR = 0.46, 95% CI = 0.31–0.67) were associated with lower odds of recent male and female partnerships. MSM in Harare who reported harassment/abuse (aOR = 3.16, 95% CI = 1.72–5.79) had higher odds of both male and female partnerships than MSM in Bulawayo reporting harassment/abuse. The prevalence of both male and female recent partnerships (31%) was lower among MSM in this survey than in other biobehavioral surveys of MSM in sub-Saharan Africa. Findings suggest that MSM with recent male and female partnerships compared to MSM with only male recent partners have lower odds of positive HIV status and participate in behaviors that lower HIV risk; however, the direction of these relationships cannot be determined due to the cross-sectional nature of the data. The findings also suggest a possible connection between experiences of stigma of MSM behavior and not having both male and female partnerships that warrants further exploration. Accessible, stigma-free HIV testing and education programming that considers the potential overlap between the MSM and general populations via both male and female partnerships and the associated behaviors could be a key component of HIV elimination in Zimbabwe.

## Introduction

Zimbabwe has a generalized HIV epidemic, with an estimated HIV prevalence of 11.9% among adults ages 15 to 49, and 1.3 million adults and children living with HIV in 2020. The state of the country’s epidemic has improved in terms of new infections, with an overall 66% decline in HIV incidence since 2010 [1]. Additionally, Zimbabwe met one out of three UNAIDS fast-track 95-95-95 targets for 2030, with 86.8% of people living with HIV (PLHIV) knowing their status, 97.0% of people who know their status receiving antiretroviral therapy (ART), and 90.3% of people on ART achieving viral load suppression as of 2020 [2].

Globally, men who have sex with men (MSM) have an increased risk of acquiring HIV compared to the general population [3–7]. In Zimbabwe, there has been limited data and focus on MSM for HIV prevention and treatment resources [3, 8, 9] despite this group’s higher prevalence of HIV (21.1%) and larger gaps in reaching HIV targets than the general population, as exemplified by estimated progress toward 95-95-95 targets reaching 73-97%-87% among MSM from Harare and Bulawayo in a 2019 bio-behavioral survey [1, 5]. Same-sex sexual activity is criminalized and penalized in Zimbabwe, and MSM face significant stigma and fear of disclosing their sexual behaviors, both socially and in healthcare settings [3, 8, 10].

In Zimbabwe, as in many high HIV burden countries, there is limited knowledge about sexual partnerships among MSM, especially MSM with both male and female sexual partnerships, and HIV risk. Previous work from sub-Saharan Africa suggests that the lack of HIV education and services for MSM combined with stigma contributes to HIV transmission by increasing hesitancy among MSM to disclose sexual behavior to their female partners [8, 11]. This could play an important role in the HIV epidemic in Zimbabwe, since MSM often face social, legal, cultural or religious pressures to marry and have children with female partners [8]. MSM with both male and female partnerships may have unique experiences with HIV risk behaviors, stigma or discrimination, and accessing HIV services compared to MSM with only male partners [11, 12], and may form a link between the epidemic within sexual networks among this key population and those of the generalized epidemic through infection of female partners [11, 13]. The possible contribution of MSM who have male and female partners to the larger overall HIV transmission networks has been examined with behavioral and genomic approaches in sub-Saharan African countries such as Kenya, South Africa, and Mozambique and suggest low or even bi-directional contribution of MSM to HIV transmission overall compared to within this key population, but studies of this type have not been conducted in Zimbabwe [14–16]. 

Using data from a 2019 biobehavioral survey conducted via respondent-driven sampling (RDS) in Harare and Bulawayo, Zimbabwe, this paper aims to describe MSM with both male and female sexual partnerships and the factors associated with such partnerships. This can contribute to understanding characteristics and sexual behaviors of MSM that may differ within the key population and better inform HIV prevention and achievement of HIV elimination in Zimbabwe.

## Methods

### Study Design and Population

A cross-sectional biobehavioral survey was conducted among MSM and transgender women (TGW) from March to July 2019 in Harare and Bulawayo, Zimbabwe. The survey methods have been described extensively elsewhere and are summarized here [5, 7]. Participant eligibility criteria included being age 18 years or older and assigned male at birth, having engaged in oral or anal sex with a man in the past 12 months, having resided in Harare or Bulawayo for at least one month, and ability to speak English, Shona, or Ndebele. Participants were also required to provide a valid recruitment coupon where applicable (see Recruitment below).

Ethical approvals were received by the Columbia University Institutional Review Board and the Medical Research Council of Zimbabwe. The protocol was also reviewed in accordance with the US Centers for Disease Control and Prevention (CDC) human research protection procedures and was determined to be research; however, CDC investigators did not interact with human participants or have access to identifiable data or specimens for research purposes.

### Recruitment

Participants were recruited using RDS, a chain-referral method of sampling that is used to produce a sample considered representative of a hard-to-reach population [17]. Recruitment chains were initiated with seed participants selected to represent a diverse range of sociodemographic characteristics and connections or influence within the MSM and TGW communities in Harare or Bulawayo. Subsequent participants were chosen via peer-recruitment. After completing the survey, each participant was given three coupons containing survey site contact information, without indicating the study’s affiliation with MSM and TGW, to distribute to peers. The number of coupons was reduced as recruitment needs slowed and then stopped when the sample size was met.

### Data Collection

Participants were asked to complete two visits to the survey offices, two weeks apart. Upon the first visit, coupons were verified and potential participants were screened for eligibility. For eligible individuals who agreed to participate, survey staff obtained written informed consent in their preferred language of English, Shona, or Ndebele. Participants could consent to complete the questionnaire without biomarker testing. The survey questionnaire administered by trained staff using a tablet captured sociodemographic information, sexual behaviors such as partnership history and condom and lubricant use, knowledge and attitudes about HIV and sexually transmitted infections, use of alcohol and drugs, engagement with HIV programs, and experiences with stigma or discrimination.

Those who consented were tested for HIV, hepatitis B (HBV), and syphilis regardless of self-reported status. Testing for HIV was performed onsite using a 3-test algorithm adapted from the Zimbabwe National HIV Testing Algorithm [18]. The first test was Alere HIV Combo (Abbott Diagnostics Scarborough, Inc., ME, USA), followed by Chembio HIV 1/2 STAT-PAK (Chembio Diagnostic Systems, Inc., Hauppauge, NY, USA), and INSTI HIV-1/HIV-2 antibody test (bioLytical Laboratories, BC, Canada). Those who tested positive for HIV received follow-up CD4 and HIV recency testing as well as viral load testing. Participants with positive test results for any of the tests were connected with appropriate care services, and individuals with negative HIV test results were provided with a referral for pre-exposure prophylaxis.

During the second visit survey staff collected unused peer coupons and assessed whether participants completed referrals to HIV and other services given at the first visit. Viral load and HIV recency test results were also provided to participants at this second visit. Participants received US$5 for each visit to the survey site and an additional US$5 for each recruited peer, up to a maximum of three.

### Measures

Survey participants were asked about their current sex or gender and their sex at birth. Participants who responded “male” to both questions were considered MSM. This data analysis was restricted to participants who were considered MSM because only 15% of 344 respondents who identified as TGW or genderqueer also reported having female partners within the past 6 months [7]. To allow for comparison of MSM with both male and female sexual partners to MSM with only male sexual partners within this time frame, the analysis was also limited to MSM who reported oral or anal sex with at least one male in the past 6 months. Participants who reported one or more male oral or anal sex partners in the previous 6 months and one or more female vaginal or anal sex partners in the previous 6 months were classified as having both male and female recent partnerships. All other MSM participants were classified as not having both male and female recent partnerships. Because the survey asked for the number of lifetime partners and the number of partners in the last 6 months, the only time period for which recency of sexual partners could be established was the last 6 months. Factors of interest were selected based on evidence found in the published literature of their association with HIV, MSM populations, or having both male and female sexual partnerships. They included participant sociodemographic information, use of HIV services, sexual behaviors and engagement in other known HIV risk behaviors, and participant-reported experiences of stigma or abuse. Marital status was defined based on participant answers when asked if they were married, cohabiting, single, separated, divorced, or widowed. Depression was assessed using the Patient Health Questionnaire-2 (PHQ-2), and participants with a score of 3 or higher were classified as likely having a major depressive disorder [19]. Hazardous alcohol use was assessed using the Alcohol Use Disorders Identification Test-Concise (AUDIT-C). A positive score indicated participants who were hazardous alcohol consumers or had active alcohol use disorders such as alcohol abuse or dependence [20, 21]. Non-injectable drug use was defined as taking non-injectable drugs or medicine for pleasure or reasons not recommended by a doctor.

The main male or main female partner was defined as the sexual partner with whom the participant had sex the most. Consistent condom use with the main male partner was defined as answering “always” when asked how often a condom was used with the main male partner in the past 6 months, consistent condom use was defined similarly for the main female partner. Participants who indicated that they had receptive anal sex the last time they had sex with their main male partner and who said that they did not use a condom the last time they had sex with their main male partner were classified as having condomless receptive anal intercourse (CRAI). Participants were asked whether they thought they might have had HIV at the time of the survey, and those who did not answer “I already know I have HIV” were asked whether they estimated their chance of becoming infected with HIV in the next 12 months as high, medium, or low.

### Data Analysis

Bivariate analyses were performed using Pearson chi-squared and Fisher’s exact tests to identify factors among MSM associated with having both male and female recent partners, and unadjusted odds ratios and corresponding 95% confidence intervals (CIs) were calculated for each variable. Variables with a *p*-value of 0.20 or less in bivariate analyses were included in multivariable logistic regression analysis. Survey city was included regardless of statistical significance based on the survey sampling approach. Knowledge of positive HIV status and HIV risk perception were excluded from multivariable analysis as they only applied to a subset of participants. Sexual identity was excluded from multivariable analysis because the statistical association between sexual identity and having both male and female recent partnerships found in bivariate analysis indicated the similarity of constructs measured by these two variables in the sample.

Variable selection for the final multivariable model was made with manual backward stepwise selection using a significance level of 0.05. Complete case analysis was used since less than 5% of data was missing. Joint tests were conducted to evaluate significant interaction between pre-specified variables. Based on evidence of interaction, an interaction term between city and experience of harassment or abuse was added to the final multivariable model. The Hosmer and Lemeshow test was used to evaluate goodness of fit of the final model. Adjusted odds ratios (aORs) were calculated and reported from the final multivariable model. The analyses were not weighted to account for sampling design because some key variables did not reach equilibrium and thus failed to meet assumptions for RDS estimators. All analyses were performed using SAS version 9.4.

## Results

### Participants Characteristics

A total of 1538 participants were enrolled into the survey. Of these, 1143 (74%) were MSM with male partners in the past 6 months and were included in the analysis. The prevalence of having both male and female recent partnerships among MSM was 31% (351/1143). The demographic characteristics of MSM in this analysis are in Table 1. Participants in both groups (with or without male and female recent partnerships) were primarily young (median age of 26) and single (recent male and female partnerships: 67% [235/351], only male recent partnerships: 83% [660/792]). A majority of participants were from Bulawayo: 54% (190/351) of those with both male and female recent partnerships and 69% (543/792) of those with only male recent partners. Almost all participants were able to both read and write (male and female recent partnerships: 99% [347/351], only male recent partnerships: 98% [778/792]) and had completed secondary or greater education (recent male and female partnerships: 95% [333/351], only male recent partnerships: 93% [740/792]). Full or part-time employment was reported by 56% (197/351) of those with both male and female recent partnerships and 46% (366/792) of those with only male recent partners. The majority of participants with both male and female recent partnerships reported bisexual identity (91%, 320/351) while the majority of participants with only male recent partners reported gay identity (76%, 598/792).

**Table 1:** Sociodemographic characteristics of MSM survey participants in Harare and Bulawayo, Zimbabwe, 2019, *N* = 1143

	n (total%)	Both Male and Female Sexual Partnerships in Past 6 Months	
		n (col%)	
		Yes (*n* = 351)	No (*n* = 792)
Age (years)			
Median (IQR)	26 (22–33)	26 (22–33)	26 (21–34)
18–24	471 (41)	136 (39)	335 (42)
25–34	426 (37)	147 (42)	279 (35)
35 and above	246 (22)	68 (19)	178 (22)
City of residence			
Bulawayo	733 (64)	190 (54)	543 (69)
Harare	410 (36)	161 (46)	249 (31)
Marital status			
Single	895 (78)	235 (67)	660 (83)
Married/Cohabiting	96 (8)	66 (19)	30 (4)
Separated/Widowed/Divorced	152 (13)	50 (14)	102 (13)
Religion			
Indicated religion^a^	917 (80)	286 (81)	631 (80)
Indicated no religion^b^	226 (20)	65 (19)	161 (20)
Highest education level			
Below secondary	70 (6)	18 (5)	52 (7)
Secondary	808 (71)	240 (68)	568 (72)
Tertiary/Vocational	265 (23)	93 (27)	172 (22)
Literacy level			
Cannot read or cannot write	18 (2)	4 (1)	14 (2)
Can both read and write	1125 (98)	347 (99)	778 (98)
Employment status			
Employed^c^	563 (49)	197 (56)	366 (46)
Unemployed	432 (38)	114 (32)	318 (40)
Other^d^	148 (13)	40 (11)	108 (14)
Sexual Identity^e^			
Gay	628 (55)	30 (9)	598 (76)
Bisexual	512 (45)	320 (91)	192 (24)
Straight/Other	2 (0)	1 (0)	2 (0)

MSM = Men who have Sex with Men. IQR = interquartile range

Percentages are rounded and may not add up to 100 for each variable

Data analysis was limited to MSM who reported at least one male oral or anal sex partner in the past 6 months

^a^Selected any one of the following religions: Traditional, Roman Catholic, Protestant, Pentecostal, Apostolic Sect, Other Christian, Muslim

^b^Answered none, did not know, or refused

^c^Employed full or part time or self-employed

^d^Responded with retired, full-time student, or other

^e^Excludes 1 participant who answered don’t know

### Sexual Behaviors, Stigma, and HIV-associated Factors

The sexual behaviors, experiences with stigma, HIV risk or risk-reduction behaviors, and HIV status of participants are shown in Table 2. Of the MSM participants, nearly all (99%, 1126/1143) consented to biomarker testing. HIV prevalence among MSM was 21% (240/1126) overall, 13% (47/351) among MSM with both male and female recent partnerships and 24% (193/792) among those with only male recent partnerships. Among the 240 participants who tested HIV-positive, 48% (114/240) overall (36% [17/47] of those with both male and female recent partnerships and 50% (97/193) of those with only male recent partners) were aware of their status.

**Table 2:** Sexual behaviors, stigma- or abuse-related experiences, known HIV-risk or risk-reduction behaviors, and HIV status among MSM in Harare and Bulawayo, Zimbabwe, 2019, *N* = 1143

	n (total %)	Both Male and Female Sexual Partnerships in Past 6 Months n (col%)	
		Yes (*n* = 351)	No (*n* = 792)
Ever tested for HIV			
Yes	972 (85)	302 (86)	670 (85)
No	171 (15)	49 (14)	122 (15)
Consistent condom use with main male partner, past 6 months			
Yes	554 (48)	183 (52)	371 (47)
No	589 (52)	168 (48)	421 (53)
Consistent condom use with main female partner, past 6 months^a,b^			
Yes	146 (42)	146 (42)	0 (0)
No	205 (58)	205 (58)	0 (0)
Condomless receptive anal intercourse at last sex with main male partner			
Yes	119 (10)	20 (6)	99 (13)
No	1024 (90)	331 (94)	693 (88)
Given money/goods for sex, past 6 months			
Yes	54 (5)	14 (4)	40 (5)
No	1089 (95)	337 (96)	752 (95)
Received money/goods for sex, past 6 months			
Yes	64 (6)	23 (7)	41 (5)
No	1079 (94)	328 (93)	751 (95)
HIV risk perception (*n* = 1032)^c^			
High	69 (7)	19 (6)	50 (7)
Medium/Low	963 (93)	314 (94)	649 (93)
Depression screening^d^			
Positive	112 (10)	31 (9)	81 (10)
Negative	1031 (90)	320 (91)	711 (90)
Alcohol use			
Yes	854 (75)	269 (77)	585 (74)
No	289 (25)	82 (23)	207 (26)
Hazardous alcohol consumption^e^			
Positive	599 (52)	204 (58)	395 (50)
Negative	544 (48)	147 (42)	397 (50)
Non-injectable drug use, past 6 months			
Yes	544 (48)	172 (49)	372 (47)
No	599 (52)	179 (51)	420 (53)
Injected drugs in past 6 months^b^			
Yes	0 (0)	0 (0)	0 (0)
No	1143 (100)	351 (100)	792 (100)
Been arrested because you have sex with men			
Yes	49 (4)	8 (2)	41 (5)
No	1094 (96)	343 (98)	751 (95)
Experienced harassment/abuse because you have sex with men			
Yes	313 (27)	64 (18)	249 (31)
No	830 (73)	287 (82)	543 (69)
Friends/family left or rejected you because you have sex with men			
Yes	147 (13)	28 (8)	119 (15)
No	996 (87)	323 (92)	673 (85)
Avoided seeking healthcare services for fear of someone finding out you have sex with men			
Yes	201 (18)	49 (14)	152 (19)
No	942 (82)	302 (86)	640 (81)
Peer educator/outreach worker talked to you about HIV			
Yes	705 (62)	209 (60)	496 (63)
No	438 (38)	142 (40)	296 (37)
HIV status			
HIV-positive	240 (21)	47 (13)	193 (24)
HIV-negative	886 (78)	300 (85)	586 (74)
Not tested	17 (1)	4 (1)	13 (2)
Aware of status among PLHIV (*n* = 240)^f^			
Yes	114 (48)	17 (36)	97 (50)
No	126 (53)	30 (64)	96 (50)

MSM = Men who have Sex with Men. PLHIV = People Living with HIV

Percentages are rounded and may not add up to 100 for each variable

Data analysis was limited to MSM who reported at least one male oral or anal sex partner in the past 6 months

^a^Among those who reported female partners in the past 6 months

^b^Not included in further analysis due to cells with 0 participants

^c^Among those who were asked about risk perception, excludes one participant who answered don’t know

^d^PHQ-2 score of 3 or higher indicates positive screening for depression

^e^AUDIT-C score of 4 or higher indicates positive screening for hazardous alcohol consumption or alcohol use disorder

^f^Participants who reported positive HIV status and received a positive result at the time of the survey

Consistent condom use in the past 6 months with their main male partner was reported by 52% (183/351) of MSM with both male and female recent partnerships, and by 47% (371/792) of those reporting only male partnerships in the past 6 months. Among those who reported having female partners in the past 6 months, 42% (146/351) reported consistent condom use with their main female partner in the past 6 months. CRAI at last sex with their main male partner was reported by 6% (20/351) of MSM with both male and female recent partnerships and 13% (99/792) of those with only male recent partnerships.

Half (50%, 395/792) of those with only male recent partnerships and slightly more than half (58%, 204/351) of those with both male and female recent partnerships were classified as hazardous alcohol consumers. Some MSM among those with both male and female recent partnerships and among those with only male recent partnerships reported ever being arrested (2% [8/351] and 5% [41/792], respectively), experiencing harassment or abuse (18% [64/351] and 31% [249/792], respectively), or having been rejected by family or friends (8% [28/351] and 15% [119/792], respectively) because they have sex with men, as well as avoiding seeking healthcare services for fear of someone finding out they have sex with men (14% [49/351] and 19% [152/792], respectively).

### Factors Associated with Having both Male and Female Partnerships

The results of the bivariate and multivariable analysis are presented in Table 3. In bivariate analyses, the following factors were associated with having both male and female recent partnerships and were eligible for inclusion in the multivariable analysis: age, survey city, marital status, highest education level, employment status, HIV status, consistent condom use with main male partner, CRAI at last sex with main male partner, hazardous consumption of alcohol, experiencing harassment or abuse because you have sex with men, having been left or rejected by friends or family because you have sex with men, having been arrested because you have sex with men, and avoiding services because of fear that someone will find out you have sex with men. Sexual identity was also significantly associated with having recent male and female partnerships but was not eligible for inclusion in multivariable analysis as described above. Complete case analysis excluded 17 participants who did not consent to biomarker testing and resulted in 1126 MSM in the final multivariable model.

**Table 3:** Bivariate and multivariable model for having both male and female sexual partnerships in the past 6 months among MSM in Harare and Bulawayo, Zimbabwe, 2019

		Bivariate			Multivariable (*N* = 1126)		
		uOR	(95% CI)	*P*-value	aOR^b^	(95% CI)	*P*-value
Age (years)^a^				0.095			0.002
18–24		1.00 (ref)	--		1.00 (ref)	--	
25–34		1.30	(0.98–1.72)		1.10	(0.80–1.52)	
35 and above		0.94	(0.67–1.33)		**0.50**	**(0.31–0.81)**	
City of residence^a^				< 0.0001			
Bulawayo		0.54	(0.42–0.70)				
Harare		1.00 (ref)	--				
Marital status^a^				< 0.0001			< 0.0001
Single		1.00 (ref)	--		1.00 (ref)	--	
Married/Cohabiting		6.18	(3.91–9.75)		**8.58**	**(4.92–14.95)**	
Separated/Widowed/Divorced		1.38	(0.95–1.99)		**1.96**	**(1.24–3.08)**	
Religion				0.479			
Indicated religion^c^		1.12	(0.82–1.55)				
Indicated no religion^d^		1.00 (ref)	--				
Highest education level^a^				0.166			
Below secondary		1.00 (ref)	--				
Secondary		1.22	(0.70–2.13)				
Tertiary/Vocational		1.56	(0.86–2.83)				
Literacy level				0.431			
Cannot read or write		0.64	(0.21–1.96)				
Can both read and write		1.00 (ref)	--				
Employment status^a^				0.009			
Employed^e^		1.00 (ref)	--				
Unemployed		0.67	(0.51–0.88)				
Other^f^		0.69	(0.46–1.03)				
Sexual identity^a,g^				< 0.0001			
Gay		1.00 (ref)	--				
Bisexual		33.22	(22.09–49.96)				
Ever tested for HIV				0.528			
Yes		1.12	(0.78–1.61)				
No		1.00 (ref)	--				
Consistent condom use with main male partner, past 6 months^a^				0.099			
Yes		1.24	(0.96–1.59)				
No		1.00 (ref)	--				
Condomless receptive anal intercourse at last sex with main male partner^a^				0.001			0.002
Yes		0.42	(0.26–0.70)		**0.43**	**(0.26–0.74)**	
No		1.00 (ref)	--		1.00 (ref)	--	
Given money/goods for sex, past 6 months				0.435			
Yes		0.78	(0.42–1.45)				
No		1.00 (ref)	--				
Received money/goods for sex, past 6 months				0.351			
Yes		1.28	(0.76–2.18)				
No		1.00 (ref)	--				
HIV risk perception among those with negative or unknown status (*n* = 1032)^h^				0.384			
High		0.79	(0.46–1.35)				
Medium/Low		1.00 (ref)	--				
Positive depression screening^i^				0.464			
Positive		0.85	(0.55–1.31)				
Negative		1.00 (ref)	--				
Alcohol use				0.320			
Yes		1.16	(0.87–1.56)				
No		1.00 (ref)	--				
Hazardous alcohol consumption^a,j^				0.010			0.002
Positive		1.39	(1.08–1.80)		**1.58**	**(1.19–2.09)**	
Negative		1.00 (ref)	--		1.00 (ref)	--	
Non-injectable drug use, past 6 months				0.526			
Yes		1.08	(0.84–1.40)				
No		1.00 (ref)	--				
Been arrested because you have sex with men^a^				0.026			
Yes		0.43	(0.20–0.92)				
No		1.00 (ref)	--				
Experienced harassment/abuse because you have sex with men^a^				< 0.0001			
Yes		0.49	(0.36–0.66)				
No		1.00 (ref)	--				
City*experienced harassment/abuse because you have sex with men^a^				< 0.0001			< 0.0001
Bulawayo/Yes		1.00 (ref)	--		1.00 (ref)	--	
Bulawayo/No		2.69	(1.75–4.13)		**2.54**	**(1.62–3.97)**	
Harare/Yes		3.08	(1.75–5.42)		**3.16**	**(1.72–5.79)**	
Harare/No		4.23	(2.71–6.62)		**3.50**	**(2.18–5.62)**	
Friends/family left or rejected you because you have sex with men^a^				0.001			
Yes		0.49	(0.32–0.76)				
No		1.00 (ref)	--				
Avoided seeking healthcare services for fear of someone finding out you have sex with men^a^				0.032			
Yes		0.68	(0.48–0.97)				
No		1.00 (ref)	--				
Peer educator/outreach worker talked to you about HIV				0.323			
Yes		0.88	(0.68–1.14)				
No		1.00 (ref)	--				
HIV status^a,k^				< 0.0001			< 0.0001
HIV-positive		0.48	(0.34–0.67)		**0.46**	**(0.31–0.67)**	
HIV-negative		1.00 (ref)	--		1.00 (ref)	--	
Aware of status among PLHIV (*n* = 240)^l^				0.083			
Yes		0.56	(0.29–1.08)				
No		1.00 (ref)	--				

aOR = adjusted odds ratio. CI = confidence interval. MSM = Men who have Sex with Men. PLHIV = People Living with HIV. uOR = unadjusted odds ratio

Bolded results are significant with 95% confidence in multivariable analysis

Data analysis was limited to MSM who reported at least one male oral or anal sex partner in the past 6 months

^a^Variable with bivariate analysis *p*-value < 0.20

^b^Adjusted for age, marital status, condomless receptive anal intercourse at last sex with main male partner, hazardous alcohol consumption, HIV status, and City^x^Experienced harassment/abuse because you have sex with men

^c^Selected any one of the following religions: Traditional, Roman Catholic, Protestant, Pentecostal, Apostolic Sect, Other Christian, Muslim

^d^Answered “none”, did not know, or refused

^e^Employed full or part time or self-employed

^f^Retired, full-time student, or other

^g^Variable recategorized for bivariate analysis, excludes 3 participants who answered straight (*n* = 2) or don’t know (*n* = 1), variable not included in multivariable analysis

^h^Among those who were asked about risk perception

^i^PHQ-2 score of 3 or higher indicates positive screening for depression

^j^AUDIT-C score of 4 or higher indicates positive screening for hazardous alcohol consumption or alcohol use disorder

^k^Among those who were tested (*n* = 1126)

^l^Participants who reported positive HIV status and received a positive result at the time of the survey

In the final multivariable model being married or cohabiting (aOR 8.58, 95% CI 4.92–14.95) or being separated/divorced/widowed (aOR 1.96, 95% CI 1.24–3.08) as compared to being single, and hazardous consumption of alcohol (aOR 1.58, 95% CI 1.19–2.09) were associated with increased odds of having both male and female recent partnerships. Being age 35 and above compared to age 18–24 (aOR 0.50, 95% CI 0.31–0.81), engaging in CRAI with the main male partner (aOR 0.43, 95% CI 0.26–0.74), and positive HIV status (aOR 0.46, 95% CI 0.31–0.67) were all associated with decreased odds of having both male and female recent partnerships. A significant interaction was found between survey city and experience of harassment or abuse. Compared with MSM in Bulawayo who experienced harassment or abuse because they have sex with men, MSM who had not experienced harassment or abuse in Bulawayo (aOR 2.54, 95% CI 1.62–3.97), MSM who had experienced harassment or abuse in Harare (aOR 3.16, 95% CI 1.72–5.79), and MSM who had not experienced harassment or abuse in Harare (aOR 3.50, 95% CI 2.18–5.62) had higher odds of having both male and female recent partnerships.

## Discussion

The prevalence of both male and female recent partnerships within the 6 months before the survey (31%) is lower among Zimbabwean MSM in this survey than in other biobehavioral surveys in sub-Saharan Africa. Surveys in Botswana in 2012 and Mali in 2015 found that 38% and 53% of MSM with male partners in the previous 6 months also had female sexual partners in the previous 6 months, respectively [22, 23]. Another study in Malawi, Botswana, and Namibia in 2008 reported a pooled prevalence of 54% among all MSM participants with lifetime history of anal sex with a male [12]. However, surveys in South Africa in 2009 and 2013 provided prevalence estimates of 2–17% [24, 25]. This suggests that there may be a wide range of engagement in both male and female sexual partnerships among MSM throughout sub-Saharan Africa, possibly based on socio-cultural differences between countries or evolving attitudes and norms at the time of data collection.

Our finding that being married or cohabiting versus being single was associated with higher odds of both male and female recent partnerships is consistent with literature from sub-Saharan Africa that reports marriage among many MSM. Some studies in the region have posed the idea that female sexual partnerships among MSM often occur due to desire or social pressure to be married and have a family [8, 12]. This is one possible explanation for the association we found between marriage/cohabitation and having recent female partners, although social pressure cannot be distinguished from bisexuality or bisexual preferences as the reason for having both male and female partnerships among MSM in this survey as participants were not asked about their motivation for seeking out female partners. Additionally, in the final multivariable model, older MSM had lower odds of having both male and female recent partners. It is difficult to determine how this finding compares to other surveys among MSM in sub-Saharan Africa. One study found no significant association between being young (less than 24 years old) and having at least one male and one female partner in the past 6 months among MSM in Namibia, Botswana, and Malawi [12]. Similarly, a survey in Nigeria reported that the association between being age 18–25 (compared to being age 26 or older) and having intercourse with both men and women in the past 2 months was not significant [11]. A paper from Kenya reported that among MSM in the study, participants who reported sex with both men and women were older than those with exclusively male partners, but the statistical association between age and having female partners was not significant [26]. Other cited analyses did not examine a direct association between age and female sexual partnerships among MSM. Marriage or cohabitation may alter the relationship between age and having both male and female partnerships such that when controlling for marital status, older MSM have significantly lower odds of having both male and female sexual partnerships. Although it is possible that marriage or cohabitation plays a role in having both male and female sexual partners, it is not clear from this analysis if other factors unrelated to marriage among older MSM explain this observation. Further exploration is needed to assess the relationship between age and having both male and female partnerships among MSM in Zimbabwe.

Stigma-related experiences such as having been arrested, rejected, harassed, or abused because of being MSM were all associated with lower odds of having both male and female recent partnerships in bivariate analyses. This is consistent with findings from a qualitative study in which MSM in Zimbabwe report facing less social stigma if they are known to have female sexual partners [8]. However, based on our data, we cannot determine whether avoiding social stigma was an explicit motivation for having female partners or a result of such activity among this sample of MSM.

The association between experiencing harassment or abuse because you have sex with men and having both male and female recent partnerships appears to vary by city. MSM who had experienced harassment or abuse because they have sex with men in Harare had higher odds of having both male and female recent partnerships than MSM who experienced harassment or abuse in Bulawayo. Further research is needed to understand whether there are social or cultural differences between cities in Zimbabwe that may contribute to an altered association between experiences of MSM-related stigma and seeking out female sexual partnerships. We cannot determine based on this data whether there is a causal relationship between experiencing harassment or abuse and having female sexual partners among MSM, only that the observed association differs by city.

CRAI was associated with lower odds of having both male and female recent partners. This aligns with a study among MSM in Namibia, Malawi, and Botswana, in which participants with both male and female recent partnerships reported higher rates of condom usage with casual and regular partners than participants with only male recent partners [12]. Additionally, we found that MSM living with HIV had lower odds of having both male and female recent partnerships. Our finding of an inverse association of both CRAI and positive HIV status with having both male and female sexual partners among MSM is consistent with other studies from sub-Saharan Africa [26–28]. A study among Kenyan MSM reported that those with only male partners were more likely to have had receptive anal sex and less likely to have had insertive anal sex than MSM who also had female sexual partners [26]. Similarly, a study in two cities in Mozambique found that a higher percentage of MSM who had only insertive anal sex with men reported both male and female sexual partners than MSM who had any receptive anal sex with men [14]. This indicates that a partial explanation for the inverse association between CRAI and having female partners may be that those with female partners less commonly engage in receptive anal intercourse. Furthermore, MSM who engage in insertive anal sex are considered to have less risk for HIV acquisition than those who engage in receptive anal sex [26, 29], which is consistent with lower odds of positive HIV status among MSM with both male and female partners compared to those with only male partners. Another explanation could be that MSM with both male and female partners may engage in HIV preventative behaviors with their male sexual partners more than MSM without female partners. Given the cross-sectional nature of the survey, it is unknown whether this is a result of female partners’ influence on MSM, a desire to engage in safer sexual behaviors given the variety of sexual partners, or MSM with recent female partners accessing better HIV education. It is also possible that some of those who know they have HIV practice risk-reducing behaviors such as using condoms consistently [30, 31] and are less likely to have female partners for reasons related to knowledge of status, including the intentional limitation of number of partners. More research is needed to explore the roles of female partners and knowledge of positive HIV status in determining sexual behaviors of MSM.

Hazardous alcohol consumption and alcohol use disorders are often considered HIV risk factors because of their association with other risks such as condomless sex; this has been documented in Zimbabwe [23, 32–34]. This association does not seem to be consistent among this sample of MSM with both male and female recent partnerships given that hazardous alcohol use was associated with having both male and female recent partnerships and those with both male and female recent partnerships have lower odds of having a positive HIV status. Literature examining factors associated with female partnerships among MSM from countries surrounding Zimbabwe lacks similar associations with hazardous alcohol consumption [12, 25, 26, 28]. There are likely other unknown factors not included in this analysis that may explain this positive association between hazardous alcohol consumption and having both male and female recent partners. For example, hazardous alcohol use and alcohol use disorders may be related to stress and mental health or other HIV-associated experiences among MSM [23, 35, 36] that cannot be fully assessed using the data from this survey.

This analysis has several limitations. Because some key variables did not reach equilibrium to meet RDS estimator conditions, the analyses were unweighted and did not account for sampling design. Additionally, the survey was conducted in only two cities in Zimbabwe and may not be representative of the entire population of MSM in Zimbabwe. The survey data is cross sectional, meaning that temporal or causal relationships between factors we examined and having both male and female partnerships cannot be determined based on findings from this analysis. While participants reported having both male and female partners in the past 6 months, we cannot determine if there was concurrency between any of these partnerships and how that may influence sexual behaviors. Further, many behaviors assessed in the survey reflect recent behaviors, but HIV may have been acquired at a time when typical behaviors were different than what was captured in the survey. All self-reported data is subject to information bias due to participant recall and social desirability biases; some behaviors recorded in the survey, especially those that may be highly stigmatized or criminalized, may not have been accurately reported by participants. The survey also had several strengths. A major strength was that it utilized RDS to recruit key populations, population groups which lack a sampling frame, especially in a setting where homosexual behavior is illegal and heavily stigmatized. This allowed a more diverse and representative sample of this key population to be recruited into the survey than would have been possible with some other sampling methods. In addition, the combination of questionnaire and biomarker data allows for a more accurate look at the relationship between attitudes or behaviors and HIV.

The results of this analysis are important in that they highlight some of the contexts in which MSM may engage in both male and female sexual partnerships and provide insight into the behaviors or experiences associated with such partnerships. More research is needed to examine the motivation for having both male and female partners and the behaviors associated with these partnerships among MSM. These behaviors and motivations are important to the understanding of how sexual networks of MSM in Zimbabwe may intertwine with the general population and play a role in transmission dynamics contributing to the generalized HIV epidemic. Other studies from sub-Saharan Africa have suggested that the contribution of MSM as a key population to the generalized HIV epidemic through the heterosexual population varies geographically and by sexual role (receptive vs. insertive). These studies have also reported that despite some evidence of HIV transmission between populations, there is a substantial amount of HIV transmission within MSM sexual networks that indicates a need for better services for this population [14–16].

MSM who participate in sexual networks that could overlap with the general population (by having both male and female partners) may engage in unique behaviors that result in a difference in HIV odds compared to MSM without female partners. HIV services in Zimbabwe that are catered specifically toward MSM or heterosexual networks separately without considering such overlap may not reach all MSM, especially given the stigmatization and criminalization of homosexual behavior. A concerted effort to ensure access to adequate, stigma-free HIV testing services and risk-reduction education programs which address both male and female sexual partnerships for MSM will be crucial to Zimbabwe’s success in achieving HIV elimination.

## Data Availability

Due to the sensitivity of the data, deidentified participant data and supporting documentation are available upon request, pending appropriate ethical and institutional approvals.
